# CD5-Positive B Lymphocytes after Kidney Transplantation

**DOI:** 10.3390/diagnostics11091574

**Published:** 2021-08-30

**Authors:** Maciej Zieliński, Agnieszka Tarasewicz, Hanna Zielińska, Magdalena Jankowska, Justyna Sakowska, Anna Dukat-Mazurek, Grażyna Moszkowska, Bolesław Rutkowski, Alicja Dębska-Ślizień, Piotr Trzonkowski

**Affiliations:** 1 Department of Medical Immunology, Medical University of Gdańsk, 80-210 Gdańsk, Poland; hzielinska@gumed.edu.pl (H.Z.); justynas@gumed.edu.pl (J.S.); adukat@uck.gda.pl (A.D.-M.); gramos@gumed.edu.pl (G.M.); ptrzon@gumed.edu.pl (P.T.); 2 Department of Nephrology, Transplantology and Internal Diseases, Medical University of Gdańsk, 80-210 Gdańsk, Poland; ataras@gumed.edu.pl (A.T.); maja@gumed.edu.pl (M.J.); bolo@gumed.edu.pl (B.R.); adeb@gumed.edu.pl (A.D.-Ś.)

**Keywords:** B cells, tolerance, biomarkers, transplantation

## Abstract

Kidney transplantation is the treatment of choice for end-stage kidney diseases. Unfortunately, kidney allograft recipients rarely develop tolerance or accommodation and require life-long immunosuppression. Among many other regulatory mechanisms, CD5+ B lymphocytes (mainly B-1a) seem to be involved in the process of allograft acceptance. These cells are the major source of natural, low-affinity antibodies, which are polyreactive. Thus, we hypothesized that CD5+ B cells could be referred to as a biomarker in those patients who developed accommodation towards kidney allotransplant. In this study, 52 low-immunized kidney transplant recipients were evaluated for transplant outcome up to 8 y post-transplant. The follow up included anti-HLA antibodies, B cells phenotype and cytokines. We have identified a cohort of recipients who produced alloantibodies (Abs+), which was associated with increased levels of CD5+ B cells, mainly during the first year after transplantation but also later on. Importantly, creatinine levels were comparable between Abs+ and Abs− allorecipients at 2 years after the transplantation and graft survival rate was comparable between these groups even eight years post-transplant. So, it seems that despite the presence of alloantibodies the graft function was sustained when the level of CD5+ B cells was increased. Targeting CD5+ B cells may be a valuable therapeutic option to increase transplant success. The phenotype can be also tried as a biomarker to increase the effectiveness of individualized post-transplant treatments.

## 1. Introduction

Kidney allotransplantation is the treatment of choice for end-stage kidney diseases. At the same time, there is ongoing research identifying accommodation and individual-recipient risk stratification. This approach may minimize immunosuppression and improve transplant outcome [1,2,3].

The successful transplant outcome relies largely on regulatory T cells (Tregs) and regulatory B cells (Bregs) secreting IL-10 [4,5]. The studies examining Bregs have identified the B cell profile of kidney-transplant recipients exhibiting operational tolerance [6]. However, intrinsic transplant tolerance is very rare. It is more commonly the accommodation that facilitates the acceptance. The accommodation can be defined as a state in which the graft is functioning despite harmful factors, e.g., alloantibodies [7]. Interestingly, up to 30% of kidney transplant recipients develop donor-specific antibodies (DSA) post-transplant without clinical signs of rejection [8]. One possible mechanism is that IgG production is skewed toward IgG2, non-complement binding antibodies and the graft survival in kidney allograft recipients positive for non-complement-binding DSA is similar to the patients without DSA [9]. Importantly, some levels of alloantibodies were required for accommodation in xenotransplant settings [10]. Interestingly, alloantibodies can be associated with accommodation also in highly sensitized kidney transplant recipients [11].

Among many Bregs subsets, B-1 cells are of special interest [12]. While B-2 cells are classical antibody-producing cells, B-1 cells are considered as a part of innate immunity because they are biased toward common motifs of bacterial antigens [13]. B-1 cells produce antibodies spontaneously, so-called natural antibodies with specificity towards bacterial antigens and autoantigens. These antibodies appear even in the absence of immunization. They are predominantly polyreactive IgM with low affinity [14,15]. Since natural antibodies bind phosphorylcholine, an antigen presented on Gram-positive bacteria, apoptotic cells and oxidized lipids, the hypothesis was raised that B-1 cells may play a role in maintaining homeostasis in the immune system [16,17]. For example, mice lacking natural antibodies develop severe autoimmune diseases [18]. B-1 cells may also regulate T cell responses. The expression of CD5 receptor on B cells promotes the production of IL-10, while upregulation of CD86 receptor leads to Th17 differentiation [19,20,21]. In-depth identification of human CD5+ B cells is difficult due to the existence of several distinct subpopulations and recent studies showed that there was a more specific phenotype for B-1 cells or Bregs [22,23]. Highlighting the complexity of B-1 cells identification, it should be noted that CD5 antigen can be expressed on B progenitors, transitional and activated B-2 cells [24,25].

This study aimed to assess the levels of CD5+ B cells in the process of accommodation in kidney allograft recipients. Despite major advances in the field, little is known how CD5+ B cells affect, if at all, the outcomes in kidney transplantation. In the mouse islet allograft rejection model, the increase in CD5+ B cell levels and elevated IL-10 concentration were noted after regulatory B cell infusion together with the clinical effect [26]. There is no such definitive data with the adoptive transfer in humans, but ABO-incompatible kidney recipients with graft loss were found to keep elevated levels of B-2 cells only [27]. CD5+ B cells were described in B-CLL patients, in whom CD5 antigen activates numerous intracellular pathways leading to the production of IL-10 [19]. Interestingly, mesenchymal stromal cells infusion in allogeneic hematopoietic stem cell transplant (HSCT) patients resulted in the increased production of IL-10 by CD5+ B cells, which was associated with clinical improvement [28].

In this study, we tried to resolve the question why post-transplant allostimulation in some patients resulted in the development of alloantibodies without clinical symptoms. Was it linked with the levels of CD5+ B cells? To address these questions we followed CD5+ B cells and other immune-tolerance markers in a cohort of kidney transplant recipients for two years post-transplant. Furthermore, the clinical outcome of the patients was followed up to eight years post-transplant.

## 2. Materials and Methods

### 2.1. Study Design

This study was a retrospective, single-centre experiment on 52 low-immunized kidney allorecipients. The detailed enrollment criteria are given in Supplementary Data. Patients were selected based on age, sex and clinical features including dialysis duration, panel-reactive antibody (PRA, complement-dependent cytotoxicity test) and human leukocyte antigen (HLA) mismatches. Patients were considered as low-immunized if CDC-PRA ≤ 20% (maximal, historical peak measurement level). We have selected low-immunized patients to study alloantibodies development and immune markers post-transplant. For this reason, the initial number of patients with alloantibodies of any specificity is low but increased during follow up (Figure S5). Upon enrollment, the first blood sample was obtained before transplantation, followed by sample collection every four months. Follow-ups occurred up to 2 y post-transplant (phase one) and continued without laboratory markers for up to 8 y (phase two) (Figure S1). The events for survival analysis were the selected clinical conditions such as graft failure or malignancy. Organ transplantation was carried out under Polish national policy for organ donation and allocation (poltransplant.org). No organs were procured from prisoners. All transplant procedures including procurement and transplantation were performed in the University Clinical Centre of the Medical University of Gdańsk, Department of Nephrology, Transplantology and Internal Diseases and Department of General, Endocrine and Transplant Surgery which cared about the privacy of the donors. Experimental protocols were approved by the local ethics committee (NKEBN, Medical University of Gdańsk, Independent Bioethics Commission for Research). All patients provided written informed consent at enrollment. For detailed methodological descriptions and a workflow schematic, see Supplementary Data.

### 2.2. Patients

Table 1 provides patient characteristics at baseline only. Subjects were divided into those who developed alloantibodies post-transplantation (Abs+) and those who did not (Abs−). This was carried out according to SPA assay-based detection of alloantibodies of any specificity at 1000 MFI cut-off. What is relevant for statistical analysis, the number of Abs+ vs. Abs− was variable at each time point, and increased from 13% (7/52) at transplantation to 37% (19/52) 2 y post-transplantation. Detailed information on alloantibodies and median MFI is given in Supplementary Data, Figure S5. Allograft failure was defined as a return to dialysis post-transplant, whereas delayed graft function (DGF) occurred when dialysis was required within the first week post-transplant. Acute tubular necrosis (ATN) was the primary cause of DGF. Polyclonal antibody (thymoglobulin) or monoclonal anti-IL-2 antibody (basiliximab) was used for induction therapy. This treatment was administrated according to the patient’s risk, such as retransplantation, a number of mismatches, high-risk donor status, etc. No individuals receiving anti-CD20 were recruited. Thymoglobulin or anti-IL-2 antibody seems to have minimal impact on CD5+ B cells [29]. Only IL-2 was reported as favourable for the induction of some Bregs-associated cells phenotype (CD27^int^/CD38+) [30]. Thus, a significantly lower rate of induction therapy in the Abs- group did not influence CD5+ B cells in this study. A standard immunosuppressive protocol was administered, comprising the following calcineurin inhibitors: ciclosporin (CsA) or tacrolimus (TAC), mycophenolate mofetil (MMF) or azathioprine (AZA) and steroids (GCs). If acute rejection developed, GCs were the therapy of choice.

### 2.3. Methods

#### 2.3.1. Flow Cytometry

Peripheral-blood B-cell subsets and Tregs were analyzed using multicolour flow cytometry in either a FACSCanto II flow cytometer (BD Biosciences, San Jose, CA, USA) or an FC500 flow cytometer (Beckman Coulter, Brea, CA, USA). CD5+ lymphocytes were identified as CD19-positive cells co-expressing the CD5 antigen. Memory B cells were CD19 cells co-expressing CD27 antigen. Regulatory T cells (Tregs) were defined as CD3+CD4+CD25+FoxP3+ cells. For details, see Supplementary Data.

#### 2.3.2. Solid-Phase Assays

The serum cytokines (TNFα, IL-1b, IL-2, IL-4, IL-6, IL-10) were determined with high-sensitivity Luminex-based multiplex assays (R&D Systems, Minneapolis, MN, USA), while BAFF and TGFβ levels were determined with ELISA (R&D Systems, Minneapolis, MN, USA). Alloantibodies were identified if MFI ≥ 1000 under the LABScreen SAB Luminex assay (OneLambda Inc, Canoga Park, CA, USA). The cut-off was in line with STAR 2017 Working Group guidelines. [31] For details, see Supplementary Data.

#### 2.3.3. Statistical Analysis

All analyses were performed in Statistica 11.0 (Statsoft, Poland). Between-group differences were determined using nonparametric tests. Significance was set at *p* < 0.05. The Kaplan–Meier estimator, log-rank tests and Gehan-Breslow-Wilcoxon were used for survival analysis. Data were expressed as medians with an interquartile range. For details, see Supplementary Data.

## 3. Results

Table S1 summarizes the primary differences in measured parameters between pre-transplantation Abs− and Abs+ patients.

### 3.1. B Cell Development

**Figure 1:**
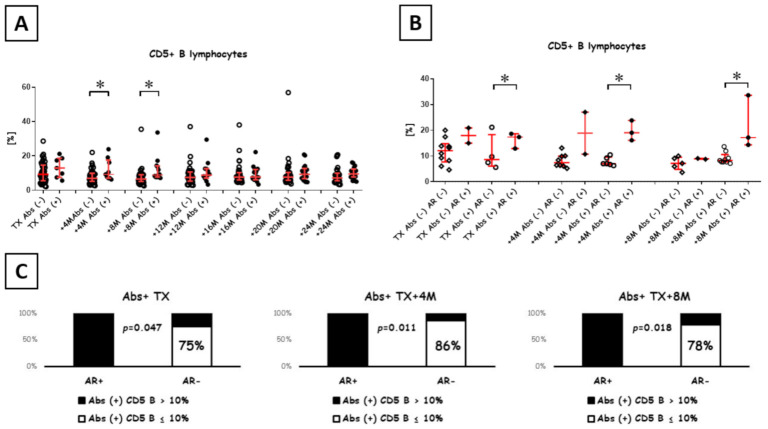
CD5+ B lymphocyte levels during the first 2 y post-transplant. (**A**), a percentage of CD5+ B lymphocytes. Open circles and black circles indicate the percent of CD5+ B cells in Abs- and Abs+ patients, respectively. The two groups were compared at each follow-up time point, starting from kidney transplant to 2 y post-transplant. (**B**), a percentage of CD5+ B lymphocytes in subgroups of Abs/AR patients limited to the transplantation, 4 and 8 months after. Data were collected from 52 patients. * indicates significant differences at *p* < 0.05 (Mann-Whitney U test). The ratio of Abs− to Abs+ recipients at measured time points was as follows: transplant (45/7), +4 months (42/9), +8 months (40/12), +12 months (37/14), +16 months (35/15), +20 months (33/17) and +24 months (32/19). * *p* < 0.05 (*U* tests). The ratio of Abs− to Abs+ recipients at measured time points was as follows: transplant (45/7), +4 months (42/9), +8 months (40/12), +12 months (37/14), +16 months (35/15), +20 months (33/17) and +24 months (32/19). (**C**), the ratio of AR incidents in Abs+ with CD5+ B cells above 10% limited to the transplantation, 4 and 8 months after.

Importantly, we observed an increase in CD5+ B cells post-transplant among Abs+ patients versus Abs- patients (Figure 1A). This difference was significant at 4 months (median 6.94% vs. 9.24%; *p* = 0.05; Mann-Whitney U test) and 8 months (median 6.50% vs. 8.88%; *p* < 0.01; Mann-Whitney U test) post-transplant. Moreover, the levels of CD5+ B cells were stable over 2 y in Abs+ patients (*p* = 0.78; Kruskal-Wallace Test). Taking into account the episodes of AR, we have noted that AR in Abs+ patients was associated with increased CD5+ B cells at 4th and 8th months post-transplant. This was not found in Abs- patients developing AR (Figure 1B). What’s more, when the cut-off of 10% CD5+ B cell was set, there were significantly more patients identified in the Abs+ population who developed AR at the points before TX, 4 months and 8 months post-transplant. The association was significant as tested by chi-square test (before TX: *p* = 0.047 and Cramer V = 0.75), (+4 months: *p* = 0.011 and Cramer V = 0.80) and (+8months: *p* = 0.018 and Cramer V = 0.68) (Figure 1C).

**Figure 2:**
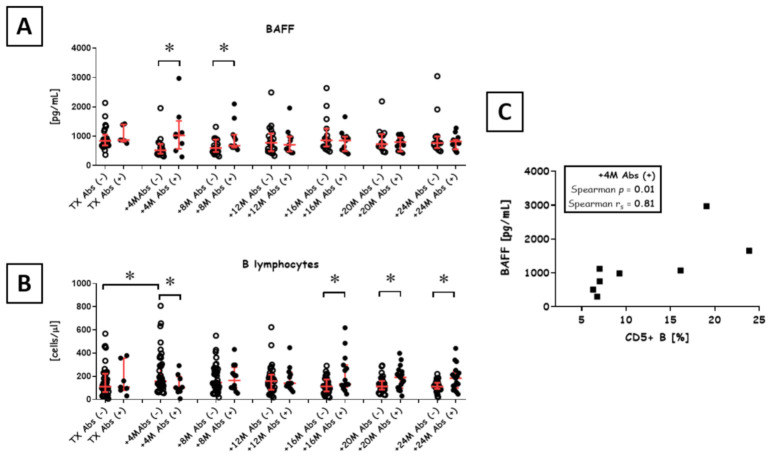
BAFF and absolute B cells levels during the first 2 y post-transplant. (**A**)**.** Serum BAFF levels in Abs− (open circles) and Abs+ (black circles) patients were compared for each time point. * *p* < 0.05 based on Mann-Whitney U tests. The ratio of Abs− to Abs+ recipients at measured time points was as follows: transplant (45/7), +4 months (42/9), +8 months (40/12), +12 months (37/14), +16 months (35/15), +20 months (33/17) and +24 months (32/19). (**B**) The correlation between BAFF (*n* = 9) and CD5+ B cells (*n* = 9) 4 months post-transplant in Abs+ recipients, as determined with Spearman rank correlation tests (r_s_, significant if *p* < 0.05). (**C**), an absolute number of cells (cells/μL). Absolute B cell frequency in Abs− (open circles) and Abs+ (black circles) patients (*n* = 52) were compared for each measured time point, from transplant up to 2 y post-transplant. * *p* < 0.05 (U tests). The ratio of Abs− to Abs+ recipients at measured time points was as follows: transplant (45/7), +4 months (42/9), +8 months (40/12), +12 months (37/14), +16 months (35/15), +20 months (33/17) and +24 months (32/19).

The total pool of B cells was stable across 2 y in Abs+ (*p* = 0.71; Kruskall-Wallace Test) but not Abs- patients (*p* = 0.01; Kruskall-Wallace Test). Among the latter, the level of B cells increased 4 months post-transplant (median 111 cells/µL vs. 158 cells/µL; *p* = 0.01; Mann-Whitney U test). Furthermore, at 4 months post-transplant, the absolute number of B cells in Abs− patients was significantly higher when compared to Abs+ patients (median 158 cells/µL vs. 102 cells/µL; *p* = 0.04; Mann-Whitney U test). However, the absolute cell numbers then increased in Abs+ patients and became significantly higher than the numbers in Abs- patients at 16 months (median 113 cells/µL vs. 131 cells/µL; *p* = 0.03; Mann-Whitney U test), 20 months (median 112 cells/µL vs. 190 cells/µL; *p* = 0.04; Mann-Whitney U test) and 24 months (median 110 cells/µL vs. 184 cells/µL; *p* = 0.02; Mann-Whitney U test) post-transplant (Figure 2C). No correlation was found between the B cell pool and BAFF, IL-10 or Tregs.

Abs− and Abs+ kidney recipients did not differ in the levels of memory (CD27+) B cells (Figure S1). As compared to the baseline, the numbers of memory B cells increased in both groups, post-transplant, in the case of Abs− patients at 12 months (median 20.83% vs. 29.81%; *p* = 0.01; Mann-Whitney U test) and in the case of Abs+ patients at 20 months (median 17.61% vs. 34.60%; *p* = 0.04; Mann-Whitney U test). The absolute cells numbers of memory B and CD5+ B were not correlated with each other.

### 3.2. Levels of B Cell-Activating Factor (BAFF) and B cells

Overall, BAFF levels increased with increasing numbers of CD5+ B cells (Figure 2). Throughout 2 y post-transplant follow up, BAFF levels were stable in Abs+ patients (*p* = 0.48; Kruskal-Wallace Test), but not in Abs− patients. In the latter group, the levels of BAFF dropped at 4 months post-transplant and were significantly lower when compared to Abs+ patients (median 519.69 pg/mL vs. 1027.54 pg/mL; *p* = 0.03; Mann-Whitney U test). The significant difference lasted up to 8 months post-transplant (median 599.33 pg/mL vs. 678.01 pg/mL; *p* = 0.04; Mann-Whitney U test) Abs+ patients exhibited also a correlation between BAFF and CD5+ B cells 4 months post-transplant (Spearman *p* = 0.01; Spearman r_s_ = 0.81). Additionally, the Abs− group exhibited a correlation between total B absolute cells number and BAFF 8 months post-transplant (Spearman *p* = 0.04; Spearman r_s_ = −0.45). No correlation was noted for BAFF and B cell pool, B naïve or B memory cells.

### 3.3. Alloantibodies (Abs)

The number of patients with the presence of allo-Abs increased from 13% (7/52) at the time of transplantation to 27% (14/52) at 12 months post-transplant (Figure S5). We recognized both defined DSA and de novo DSA. By 2 y post-transplant, the percentage of patients with confirmed Abs increased to 37% (19/52). Collective data on the number of alloantibodies and MFI levels per patient are given in Figure S5. Additionally, C1Q assays demonstrated that only one recipient had complement-binding alloantibodies persisting up to 2 y post-transplant. In general, IL-4 levels were comparable across the study for both Abs− and Abs+ patients (Kruskal-Wallace Test: *p* = 0.88; *p* = 0.91, respectively). Between-group significant difference occurred at long-term with more IL-4 in Abs+ than in Abs− patients (Mann-Whitney U test: at 12 months: median 75.12 pg/mL vs. 35.88 pg/mL, *p* = 0.01; at 20 months: median 66.32 pg/mL vs. 38.37 pg/mL, *p* = 0.02; Figure S6).

### 3.4. Anti-Inflammatory Cytokines

**Figure 3:**
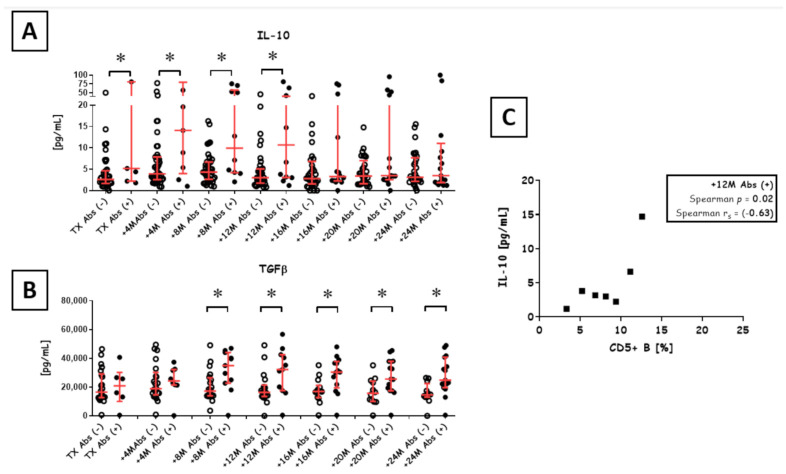
Anti-inflammatory cytokines during the first 2 y post-transplant. Serum IL-10 (**A**) and TGFβ (**B**) levels in Abs- (open circles) and Abs+ (black circles) were compared for each measured time point across 52 patients. * *p* < 0.05 based on Mann-Whitney U tests. A correlation between IL-10 and CD5+ B cells among Abs+ patients at 12 months post-transplant (**C**). The ratio of Abs− to Abs+ recipients at measured time points was as follows: transplant (45/7), +4 months (42/9), +8 months (40/12), +12 months (37/14), +16 months (35/15), +20 months (33/17) and +24 months (32/19).

As compared to Abs- patients, Abs+ patients were characterized by higher levels of anti-inflammatory cytokines (IL-10 and TGFβ) (Figure 3). Levels of both cytokines were comparable over time in Abs+ patients for up to 2 y (IL-10: *p* = 0.40; TGFβ: *p* = 0.70; Kruskall-Wallace Test), whereas only TGFβ was stable in Abs− patients (*p* = 0.63; Kruskall-Wallace Test). IL-10 levels were higher in Abs+ than in Abs− patients at 4 months (median 14.07 pg/mL vs. 3.94 pg/mL; *p* = 0.04; Mann-Whitney U test), 8 months (median 9.93 pg/mL vs. 4.31 pg/mL; *p* = 0.03; Mann-Whitney U test) and 12 months (median 10.67 pg/mL vs. 3.19 pg/mL; *p* = 0.02; Mann-Whitney U test). Interestingly, at 1 y post-transplant, serum concentrations of IL-10 decreased. We also confirmed a correlation between IL-10 and CD5+ B cells among Abs+ patients at 12 months post-transplant (Spearman *p* = 0.02; Spearman r_s_ = 0.67). The same correlation was found in Abs− patients at 20 months post-transplant (Spearman *p* = 0.03; Spearman r_s_ = −0.63).

### 3.5. Pro-Inflammatory Cytokines

**Figure 4:**
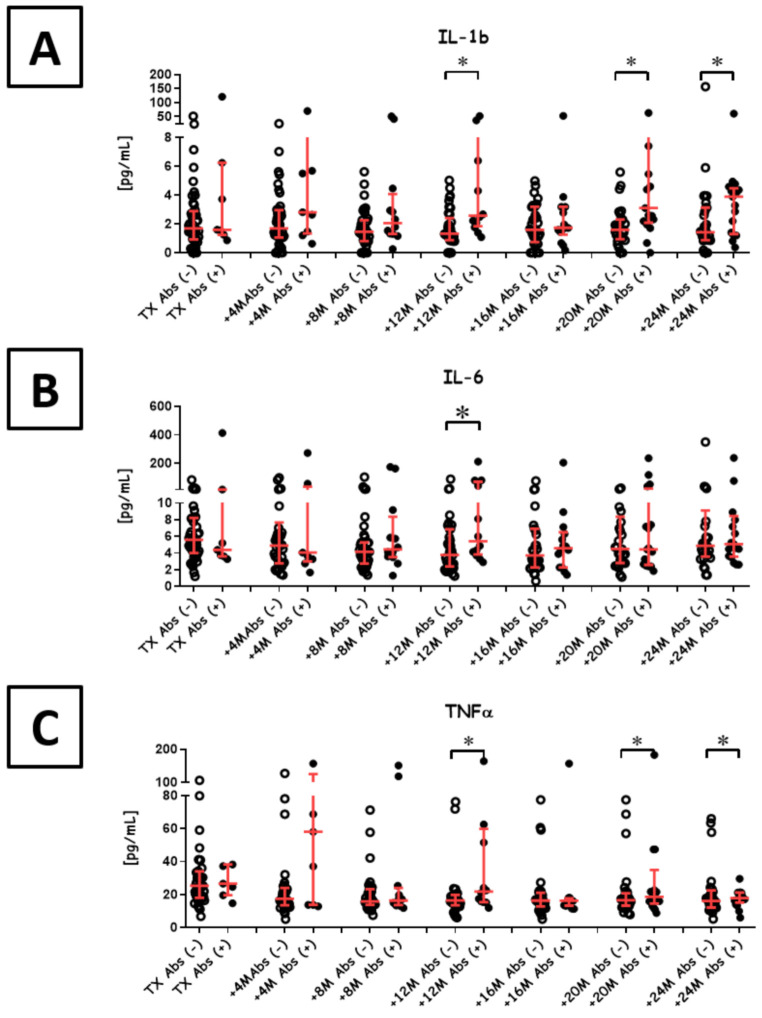
Pro-inflammatory cytokines during the first 2 y post-transplant. Serum IL-1b (**A**), IL-6 (**B**) and TNFα (**C**) levels in Abs− (open circles) and Abs+ (black circles) patients were compared across for each measured time point. * *p* < 0.05 based on Mann-Whitney U tests. The ratio of Abs− to Abs+ recipients at measured time points was as follows: transplant (45/7), +4 months (42/9), +8 months (40/12), +12 months (37/14), +16 months (35/15), +20 months (33/17) and +24 months (32/19).

Pro-inflammatory cytokines in Abs+ patients did not differ over time (IL-1b: *p* = 0.83, IL-6: *p* = 0.11, TNFα: *p* < 0.01; Kruskal-Wallace Test). In general, Abs+ patients were characterized by slightly higher IL-1b, IL-6 and TNFα levels than Abs- patients (Figure 4). Abs- patients kept sustained levels of IL-1b for 2 y (*p* = 0.11; Kruskal-Wallace Test), whereas TNFα and IL-6 were consistently decreasing until 16 months post-transplant. After this time point, IL-1b, IL-6 and TNFα returned to the baseline levels in this group. Between groups comparisons revealed significantly lower levels of all three cytokines in Abs- patients, when compared to Abs+ ones (IL-1b: median 1.32 pg/mL vs. 2.58 pg/mL, *p* < 0.01; Mann-Whitney U test; IL-6: median 3.77 pg/mL vs. 5.43 pg/mL, *p* = 0.02; Mann-Whitney U test; TNFα: median 16.42 pg/mL vs. 21.74 pg/mL, *p* = 0.04; Mann-Whitney U test). There was no correlation between CD5+ B cells and the levels of IL-1b, IL-6 or TNFα.

### 3.6. Regulatory T Cell Levels

**Figure 5:**
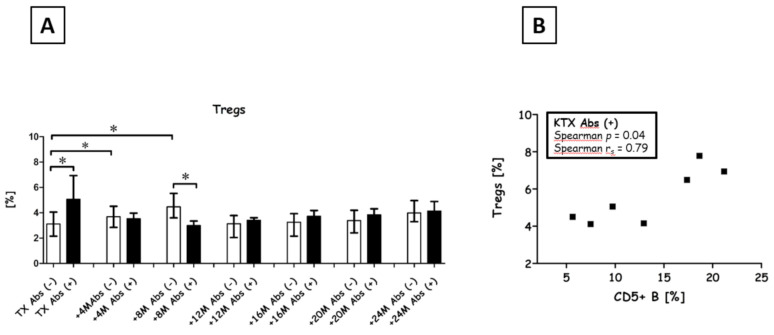
Regulatory T cells during the first 2 y post-transplant. (**A**) Treg levels in Abs− (open circles) and Abs+ (black circles) patients were compared for each measured time point. * *p* < 0.05 based on Mann-Whitney U tests. The ratio of Abs− to Abs+ recipients at measured time points was as follows: transplant (45/7), +4 months (42/9), +8 months (40/12), +12 months (37/14), +16 months (35/15), +20 months (33/17) and +24 months (32/19). (**B**) Correlation between Tregs (*n* = 7) and CD5+ B cells (*n* = 7) before transplant in Abs+ recipients, as determined with Spearman rank correlation tests (r_s_, significant if *p* < 0.05).

We observed a variable levels of Tregs among Abs+ recipients (*p* < 0.01; Kruskall-Wallace Test; Figure 5). Among Abs− patients, Treg levels increased at 4 months (median 3.69% vs. 3.11%, *p* = 0.04; Mann-Whitney U test) and 8 months (median 4.47% vs. 3.11%, *p* < 0.01; Mann-Whitney U test) post-transplant. Additionally, Treg levels differed significantly between Abs− and Abs+ patients both before transplant (median 3.11% vs. 5.06%; *p* < 0.01; Mann-Whitney U test) and 8 months after (median 4.47% vs. 2.99%; *p* < 0.01; Mann-Whitney U test). Interestingly, Treg and CD5+ B cell frequencies were correlated in Abs+ patients before transplantation (Spearman *p* = 0.04, Spearman r_s_ = 0.79).

### 3.7. Transplant Outcome

**Figure 6:**
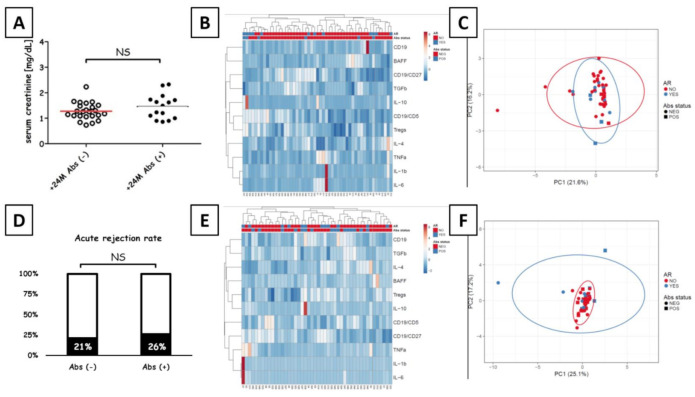
Kidney transplant outcome after 2 y. (**A**) Serum creatinine levels in Abs− and Abs+ measured for 2 y post-transplantation in the study population (*n* = 52). Clustering and heat map analysis of patient profiles at transplantation (**B**) and 2 y later (**E**) did not reveal significant differences for either time point based on Euclidean distances between clusters. Principal component analysis on all parameters measured in Abs− and Abs+ kidney recipients, before (**C**) and 2 y after (**F**) the transplant. The analysis included 45 Abs− and 7 Abs+ recipients at transplant, along with 32 Abs- and 17 Abs+ recipients 24 months later. Unit variance scaling was applied to rows; SVD with imputation was used to calculate principal components. Respectively, principal components 1 and 2 explain 21.6% and 16.2% of total (pre-transplant variance, as well as 25.1% and 17.2% of total variance 2 y post-transplant (*n* = 52 for both time points). A new observation from the same group will fall inside the prediction ellipse with a 0.95 probability. (**D**) The proportion of patients developing AR (in black) among the study population (*n* = 52). Chi-squared tests revealed no significant differences (NS, not significant, *p* > 0.05). At 2 y post-transplant, the study included 32 Abs− patients and 17 Abs+ patients.

**Figure 7:**
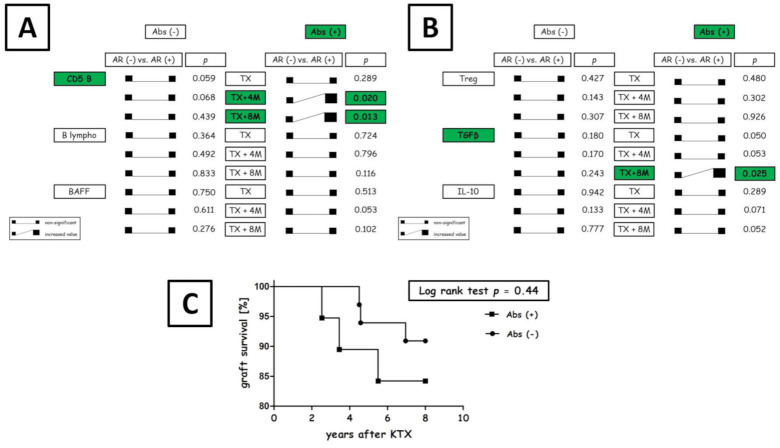
Graft survival rate at 8 y post-transplant. (**A**,**B**), the differences in CD5+ B cells, total B cell count, Tregs, IL-10, TGFβ and BAFF across Abs− and Abs+ in respect to the acute rejection incidents limited to the transplantation, 4 and 8 months after. (**C**), graft survival rate was 90% for Abs− (*n* = 33) and 84% for Abs+ (*n* = 19) recipients. This difference was not significant based on both the log-rank test (*p* = 0.44) and the Gehan-Breslow-Wilcoxon test (*p* = 0.41). The hazard ratio was 0.51 (95% CI: 0.09–2.76).

Kidney rejection rates did not differ between Abs- and Abs+ recipients (*p* = 0.67, Cramer V = 0.06). The episodes of AR were confirmed in 7 out of 33 (21%) Abs− patients and in 5 out of 19 (26%) Abs+ patients (median 3.11% vs. 5.06%, *p* < 0.01; Mann-Whitney U test). Abs− patients who developed AR (7/33) were diagnosed with acute cellular rejection (ACR). The anti-donor antibody-mediated rejection (ABMR) was diagnosed in two Abs+ recipients (2/5) and 3 Abs+ patients who developed ACR (3/5). At 2 y post-transplant, Abs+ and Abs− recipients were characterized by similar serum creatinine levels (median 1.49 mg/dl vs. 1.26 mg/dl, *p* = 0.24; Mann-Whitney U test; Figure 6). Furthermore, DGF rates were comparable between the groups (*p* = 0.79; Cramer V = 0.04; Figure S7). Graft survival was also comparable between the groups as long as 8 y post-transplant (log-rank test, *p* = 0.44) (Figure 7C); Likewise, between-group patient mortality did not differ up to 8 y post-transplant (log-rank test, *p* = 0.53; Figure S8). The only malignancy found in comparable rates across the two patient groups over 8 y was skin neoplasm (non-melanoma skin cancer) (Figure S9). Finally, we have compared CD5+ B cells, total B cell count, Tregs, IL-10, TGFβ and BAFF across Abs− and Abs+ in respect to the acute rejection incidents. Once the parameters were compared between acute rejection (AR+) and non-acute rejection (AR-) groups for both Abs− and Abs+, we have found some important differences. Those Abs+ patients that developed AR had increased CD5+ B cells at 4th and 8th month after transplantation. Similarly, the TGFβ was elevated eight months posttransplant as compared to non-rejection patients (Figure 7A,B).

## 4. Discussion

Despite immunosuppressive treatment after kidney transplantation, there is a gradual and inevitable immune response to the HLA mismatched antigens resulting in the generation of alloantibodies. In this study, we demonstrated that increased frequencies of CD5+ B cells and elevated IL-10 concentration can be found in the recipients who developed alloantibodies post-transplant. Furthermore, we have found that acute rejection was associated with CD5+ B cells levels above 10% cut-off (Figure 1). What’s more, related to low-immunized patients, the risk of kidney rejection was comparable up to 8 y follow-up (Figure 7). To our knowledge, this is the first study analyzing the association between CD5+ B cells and long-term clinical outcomes after kidney transplantation. The scientific significance of our study lies in the demonstration that the balance between CD5+ B and CD5- B cells along with pro-tolerogenic factors are potentially a part of the accommodation process to a newly transplanted organ. Here, the accommodation is defined as acquired resistance to immune-mediated rejection despite the presence of alloantibodies [7].

We did observe that Abs+ patients kept more CD5+ B cells than Abs− patients, notably during the first year post-transplant. Previous reports on CD5+ B cells in ABO-incompatible transplantations showed that kidney rejection was associated with an early reduction in the level of CD5+ B cells [27]. Contrary, it has been reported that sustained graft function after withdrawal of immunosuppression was associated with a high level of CD5+ B cells [32]. Another line of evidence comes from autoimmune models such as ANCA-associated vasculitis. Here, patients depleted with rituximab with a low proportion of CD5+ B cells during repopulation were at risk of early relapse [33]. Collectively, our findings and previous research demonstrate that CD5+ B cells and IL-10 might promote long-term accommodation towards allograft as long as 8 y post-transplant. This clinical study confirms that CD5+ B cells in humans along with Tregs can be attributed to the accommodation [4,34]. Interestingly, a similar correlation between IL-10 and Bregs was previously noted in systemic lupus erythematosus patients [35].

Another notable finding was the lack of an increase in the levels of memory B (CD19+CD27+) cells for up to 20 months post-transplant (Figure S4). We observed an increase in BAFF among Abs+ patients that the most likely promoted CD5+ B cell production (Figure 2) and there was a strong positive correlation between the levels of BAFF and the numbers of CD5+ B cells (*p* = 0.01, Spearman r_s_ = 0.81) at 4 months post-transplant. This observation suggests that B cell depletion may be in some aspects an inadequate therapy, as it reduces CD5+ B cell compartment. As previously shown, B cell depletion (anti-CD20) as an induction therapy may increase the rate of ACR, probably due to Bregs depletion [36].

Interestingly, we found also an increase in the levels of IL-10 concordant to the levels of alloantibodies among Abs+ recipients (Figure 3A). Additionally, IL-10 was elevated in Abs+ patients even before transplant surgery. One possible explanation for this correlation could be the elevated level of Tregs in Abs+ patients, which was the only parameter making them different from Abs− patients before transplantation (Table S1). It is not clear why end-stage renal disease patients (ESRD) keep more Tregs than healthy individuals. The recent reports show that Tregs in ESRD are highly activated and express GARP (glycoprotein A repetitions predominant) which can boost the level of these cells at the periphery. The expression of GARP could be also an indirect hallmark of alloreactivity, which may partially explain Tregs mobilization and activation due to immunization incidents in ESRD patients (i.e., blood transfusions) [37].

We have also observed a kind of switch from IL-10 to TGFβ among Abs+ recipients 1 y post-transplant (Figure 3). Importantly, TGFβ produced by the regulatory B cells may promote Tregs toward graft survival [38]. During this period, the levels of pro-inflammatory cytokines also increased (Figure 4). A recent autoimmune model using metabolic signals revealed synergistic cooperation between IL-10 and TGFβ in the suppression of B cells [39]. This may partially explain why we observed only one recipient who persistently displayed complement-binding IgG with DSA specificity. Nevertheless, this cytokine milieu is full of different signals as Abs+ patients exhibited also slightly increased levels of IL-4 (Figure S6). This cytokine, together with IL-21, consist of an essential signal that stimulates B cells to IgG switching and promotes the IgG1 subset. IgG1 is a complement-binding immunoglobulin that likely plays a role in complement-dependent cytotoxicity [40]. Importantly, patients with non-complement-binding DSA and patients without DSA have similar graft survival [9].

The number of CD5+ B cells may be affected by many factors. One possible mechanism is ageing. Indeed, CD5+ B cells are dominant during the neonatal period but they undergo substantial reduction throughout the ontogeny [41]. This occurs as these cells develop from distinct progenitors that are less abundant in the postnatal bone marrow [42]. However, this factor in our study seems to be negligible as Abs- and Abs+ groups did not differ significantly in age (Table 1).

Our results will be important for clinical trials using adoptive cell therapy in transplantation. CD5+ B cells might be a valuable target for the development of novel therapies to improve long-term outcomes after kidney transplantation. In animal models of islet allograft rejection, regulatory B cells infusion resulted in the immunomodulatory effects mediated by CD5+ cells and IL-10 [26]. In humans, Bregs were suggested as prophylaxis of graft versus host disease (GVHD) after allogeneic hematopoietic stem cell transplantation (HSCT) [43]. Another idea is to use mesenchymal stromal cells infusion to induce IL-10 production by CD5+ B cells in HSCT patients [28]. Nevertheless, there is a potential issue confronting the use of CD5+ B cells as a therapeutic tool. There are reports that these cells are involved in different pathogenic conditions such as IgA nephropathy or malignancies, especially chronic B lymphocyte leukaemia or mantel cell lymphoma [44,45]. Nonetheless, our long-term observations did not identify any signs of B cell malignancy. The only notable side-effect was the appearance of skin neoplasms in both Abs- and Abs+ (Figure S9).

Our study has some limitations. First, we identified relatively few patients who developed alloantibodies despite examining a large subject pool, 13% (7/52) at the time of transplantation and 37% (19/52) 2 y post-transplant. The selection of low-immunized individuals probably explains this outcome (0% PRA at TX and maximum 20% PRA historical peak). In this study, an increase in the number of Abs+ recipients was noted at 12 and 24 months post-transplant, reaching up to 37% of all individuals (Figure S5). One of the possible reasons for this increase might be a change in immunosuppressive drugs. However, neither group exhibited any differences in the serum concentrations of tacrolimus nor cyclosporine between 1 and 2 y post-transplant (Figure S10). It would be also interesting to study CD5+ cells in the context of anti-idiotypic antibodies and to include patients with unstable graft function.

In conclusion, our data suggest that CD5+ B cells are an important factor associated with the prognosis of graft success in kidney transplantation an increased level of CD5+ B cells along with IL-10 may guide clinically the development of accommodation post-transplant despite alloantibodies present. These findings encourage future research on B lymphocytes and graft survival. Moreover, our data should be translated to the clinic to individualize immunosuppressive protocols after kidney transplants.

## Figures and Tables

**Table 1:** Pre-transplant patient’s characteristics.

Recipient Pre-Transplant Status	Abs−	Abs+	*p* **
Age (years, mean ± SD)	53 ± 11	52 ± 14	0.64
Sex (male/female)	27/18	3/4	0.39
Cause of kidney failure glomerulonephritis diabetes mellitus hypertensive nephropathy autoimmune (SLE, anti-GBM) other *			
	11	4	NT
	7	0	NT
	5	0	NT
	4	0	NT
	8	3	NT
Dialysis (months; median)	11	59	0.02
1st transplantation	42	4	NT
Retransplantation	3	3	NT
HLA mismatches: A/B/DR (median; range)	3 (1–6)	4 (2–5)	0.60
locus A	1	2	NT
locus B	2	1	NT
locus DR	1	1	NT
HLA mismatches from previous KTX: A/B/DR (median; range)	2 (2–3)	3 (3–4)	NT
Immunization			
pregnancies	12	3	NT
blood products transfusion	6	1	NT
PRA (panel reactive antibodies) before TX historical peak/last (median)	18/0	8/0	NT
historical (range)	6–20	0–10	NT
last (range)	0-3	0-6	NT
Donor status			
deceased	43	5	NT
living	2	2	NT
CMV status (number of positive, %)	21 (47%)	3 (43%)	0.57
Induction therapy (total, % of group) polyclonal antibody (thymoglobulin) monoclonal anti IL-2 antibody (basiliximab)	6 (13%)	5 (71%)	0.03
	1	2	NT
	5	3	NT
Immunosuppressive regimen			
MMF/CsA/GCs	11	1	0.55
MMF/TAC/GCs	34	6	0.55

* autosomal dominant polycystic kidney disease, rapidly progressive glomerulonephritis, hereditary nephritis or unknown; ** χ^2^ test of association or Mann-Whitney U-test comparing Abs- vs. Abs+; NT, not tested; the total number of patients (*n* = 52), Abs+ (*n* = 19) and Abs− (*n* = 33).

## Data Availability

The datasets generated during the current study are available from the corresponding author on reasonable request.

## Supplementary Materials

The following are available online at https://www.mdpi.com/article/10.3390/diagnostics11091574/s1, Supplementary Materials. Patients and enrollment criteria; Figure S1. Study design; Table S1. Initial differences in measured parameters between Abs- and Abs+ recipients before transplantation; Flow cytometry methods; Figure S2. A representative example of B lymphocyte gating; Figure S3. A representative example of Treg gating; Other laboratory methods; Statistics; Supplementary results section; Figure S4. Memory B lymphocyte frequency at 2 y post-transplant; Figure S5. Number of recipients developing alloantibodies at 2 y post-transplant and a table with a number of alloantibodies and MFI levels in each recipient; Figure S6. IL-4 levels at 2 y post-transplant; Figure S7. Rate of delayed graft function (DGF); Figure S8. Patient survival rate at 8 y post-transplant; Figure S9. Neoplasm rate at 8 y post-transplant; Figure S10. Serum concentrations of tacrolimus and cyclosporine after transplantation.
