# Supplementary material for: CD5-Positive B Lymphocytes after Kidney Transplantation
Source: Diagnostics (Basel). 2021 Aug 30;11(9):1574. doi: 10.3390/diagnostics11091574 (PMC8470969; doi:10.3390/diagnostics11091574)
Supplement: Supplementary file 1 [file diagnostics-11-01574-s001.zip › diagnostics-1326160-supplementary.pdf]

CD5 Positive B Lymphocytes Dynamics After Kidney Transplantation – Supplementary data

Maciej Zieliński, MSc, PhD;[1] Agnieszka Tarasewicz, MD, PhD;[2] Hanna Zielińska, MSc, PhD;[1] Magdalena Jankowska, MD, PhD;[2] Justyna Sakowska, MSc;[1] Anna Dukat-Mazurek, MSc, PhD;[1] Grażyna Moszkowska, MSc, PhD;[1] Alicja Dębska-Ślizień, MD, PhD;[2] Bolesław Rutkowski, MD, PhD;[2] and Piotr Trzonkowski, MD, PhD[1]

[1] Department of Medical Immunology, Medical University of Gdańsk, Poland

[2] Department of Nephrology, Transplantology and Internal Diseases, Medical University of Gdańsk, Poland

Materials and methods

Patients

A schematic of the study procedures is presented in Figure S1. Patients received kidney transplants according to the nationwide organ allocation policy, based on a negative CDC crossmatch (CDC-XM).[1] In each case, CDC-XM was performed separately for the entire lymphocyte pool and for the B cell population. Only those ≥18 y old were eligible for the study. Participants were excluded if they underwent induction or treatment with B cell depletion therapy. Detailed enrollment criteria were as follows:

1. GENERAL CRITERIA:

1.1. Informed consent of patient

1.2. Patient age

Middle-aged (median: 50 years) kidney recipients

No statistical differences in age between subgroups

1.3. Patient sex

Both males and females

No statistical differences in number of males/females between subgroups.

1.4. Number of patients

Minimum 40, maximum 60

1.5. Type of transplantation

Only kidney transplant (primary and re-transplant) recipients; no multiorgan transplantation

2. DONOR/RECIPIENT HCMV SEROSTATUS:

2.1. Both infected/non-infected donor/recipient pairs

2.2. Prevalence of R+ (D+/R+ and D-/R+) patients, but no strict minimal-numbers criteria

3. CLINICAL CRITERIA:

3.1. Cause of kidney failure, no strict criteria

3.2. Dialysis period before transplantation

No longer than 5 y before transplantation

3.3. HLA immunization status

Statistically comparable median numbers of HLA mismatches between subgroups

Low immunization status according to PRA (CDC) before transplantation and historical, up to 20%

No statistical differences in PRA level between subgroups

Minimum time brake (4 weeks) from immunization incident

3.4. Deceased/living donors, no strict criteria

Figure S1. Schematic of study procedures

All kidney recipients were subjected to a standard triple immunosuppression protocol. This comprised calcineurin inhibitors (cyclosporine-CsA or tacrolimus-TAC), mycophenolate mofetil (MMF) or azathioprine (AZA), and steroids (GCs). Experienced nephrologists confirmed kidney rejections using clinical and laboratory markers (graft biopsy-Banff grade, Doppler investigation, de novo alloantibodies-SPA, serum creatinine, and patient symptoms).

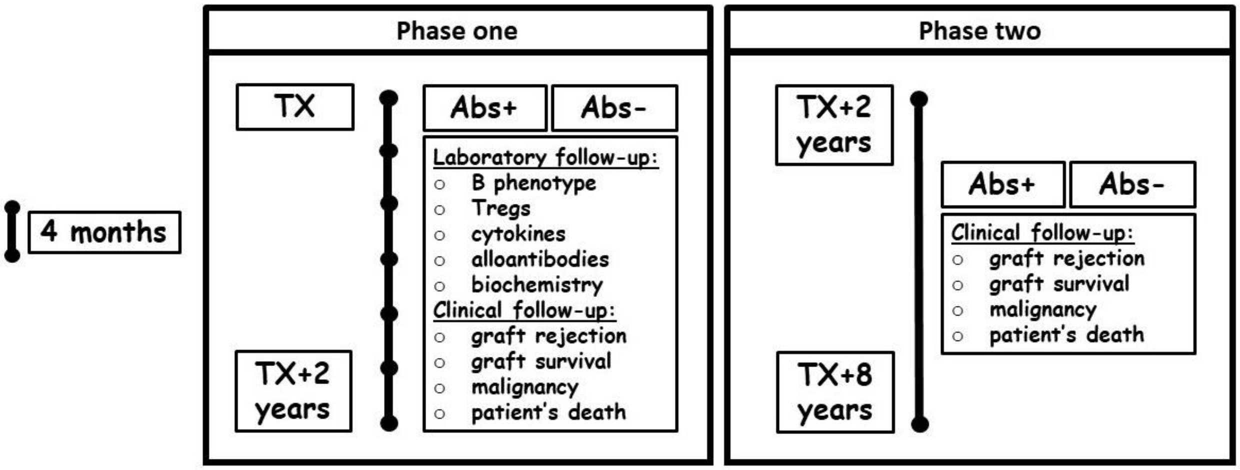

Flow cytometry

Flow cytometry was performed using freshly obtained EDTA whole-blood samples. Whole blood was stained with a monoclonal antibody cocktail: CD19 ECD clone J3-119, CD5 PE clone BL1a, and CD27 PC5 clone 1A4CD27. Cells were counted using Flow-Count Fluorospheres (Beckman Coulter, Brea, CA). For the regulatory T cell (Treg) assay, a peripheral blood mononuclear cell (PBMC) was obtained using Ficoll Paque Plus density-gradient media (GE Healthcare) and stained with CD3 Pacific Blue clone UCHT1, CD4 PerCP clone SK3, and CD25 PE clone M-A251 (BD Biosciences, San Jose, CA). Cells were then permeabilized with Foxp3/Transcription Factor Staining Buffer Set and stained with FoxP3 APC clone 236A/E7 (Thermo Fisher Scientific, Waltham, MA), as previously described.[2,3,4] For sample readout, either a FC500 (Beckman Coulter) or a FACSCanto II (BD Biosciences) flow cytometer was used. Subsequent analysis was performed in Kaluza version 1.2 (Beckman Coulter). Representative examples of lymphocyte gating are provided in Figures S2 and S3.

Figure S2. Representative example of B lymphocyte gating

(A) An forward scatter (FSC) vs. side scatter (SSC) cytogram was used to exclude debris and gate all leukocyte populations. **(B)** An SSC vs. CD19 cytogram was then created, and B lymphocytes were gated as SSC low/CD19 positive. **(D)** Using "Lympho B" gate, CD27 expression was assessed, and memory B cells were identified as CD19/CD27 double-positive B cells. **(F)** CD19/CD5 double-positive cells were identified as CD5+ B lymphocytes. **(C)** Beads for absolute cell counting were identified using ECD fluorescence channel. The population of interest was gated with the fluorescence minus one approach; the FMO bound for CD19/CD27 **(E)** and CD19/CD5 **(G)**. In each run, 75.000 events were collected.

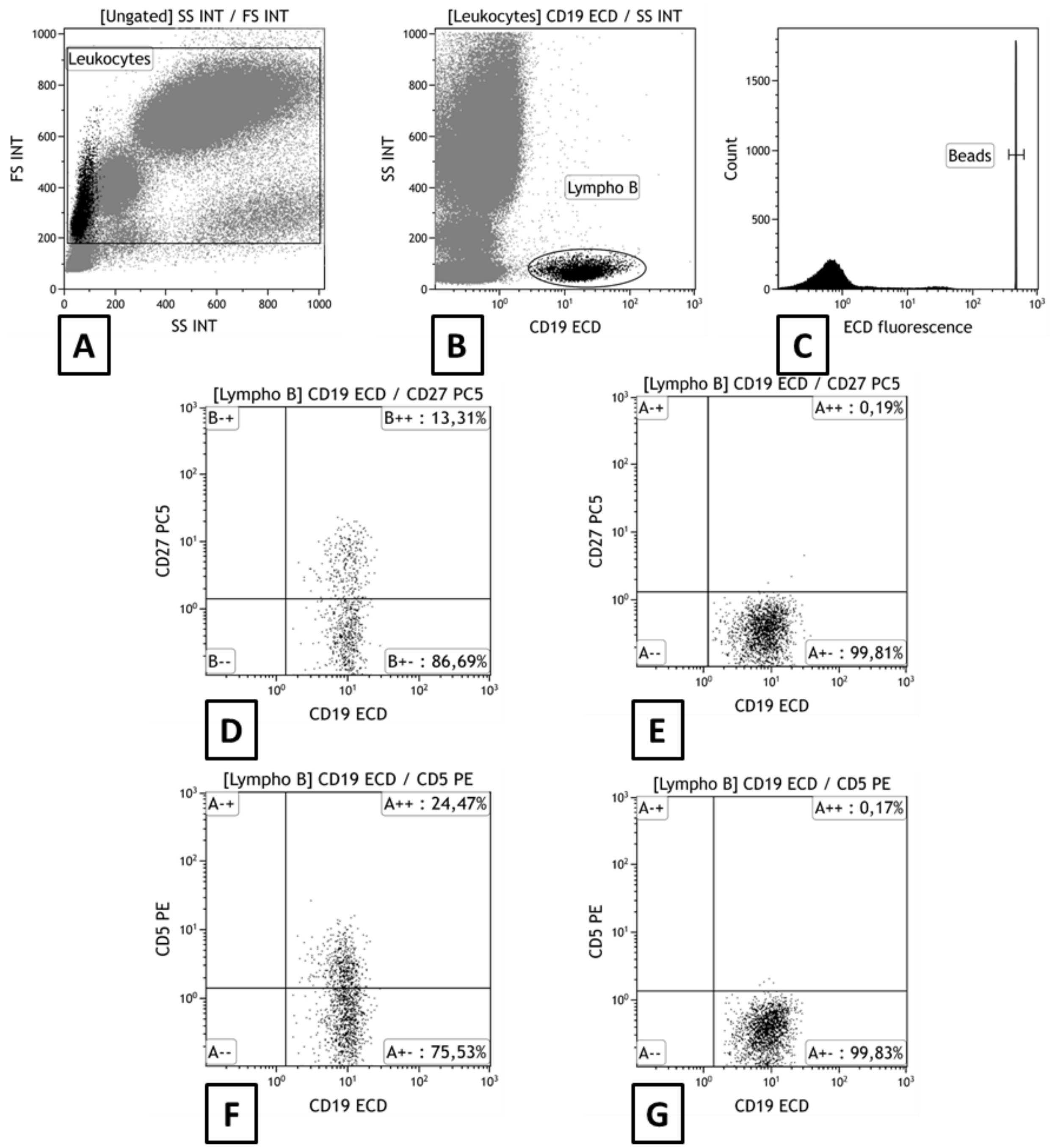
[Ungated] SS INT / FS INT
Leukocytes
FS INT
SS INT
A
[Leukocytes] CD19 ECD / SS INT
Lympho B
SS INT
CD19 ECD
B
Beads
Count
ECD fluorescence
C
[Lympho B] CD19 ECD / CD27 PC5
B-+
B++ : 13,31%
B--
B+- : 86,69%
CD27 PC5
CD19 ECD
D
[Lympho B] CD19 ECD / CD27 PC5
A-+
A++ : 0,19%
A--
A+- : 99,81%
CD27 PC5
CD19 ECD
E
[Lympho B] CD19 ECD / CD5 PE
A-+
A++ : 24,47%
A--
A+- : 75,53%
CD5 PE
CD19 ECD
F
[Lympho B] CD19 ECD / CD5 PE
A-+
A++ : 0,17%
A--
A+- : 99,83%
CD5 PE
CD19 ECD
G

Figure S3. Representative example of Treg gating

(A) The "Singlets" gate was created after doublets were excluded. **(B)** Lymphocytes were gated as FSC vs. SSC. **(C)** CD4 T lymphocytes were gated as CD3/CD4 double-positive cells. **(D)** The "Lympho T CD4" gate was used to assess CD25 surface antigen and intracellular FoxP3 expression, identifying Tregs as CD25high/FoxP3 double-positive CD4 T cells. Appropriate gating was applied using the fluorescence minus one approach. In each run, 100.000 events were collected.

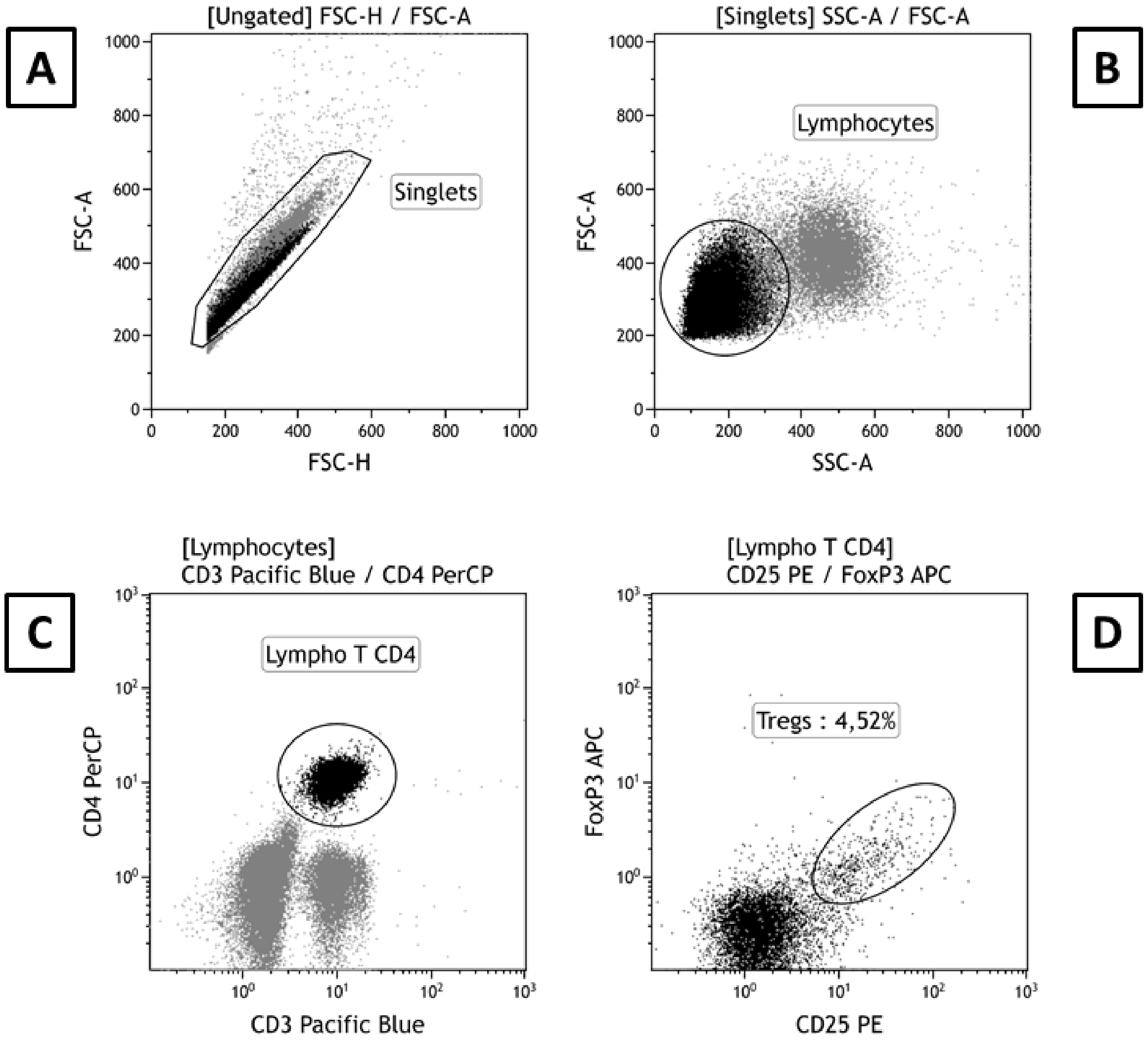

Cytokines

Luminex multiplex assay

Serum samples were collected and frozen at -80°C, then thawed only once before cytokine analysis. TNFα, IL-1b, IL-2, IL-4, IL-6, and IL-10 were quantified using a multiplex assay, based on a standard Luminex kit (Base Kit, High Sensitivity Cytokine Panel A, R&D Systems, Minneapolis, MN) supplemented with high-sensitivity kits. Samples were analyzed following manufacturer protocol on a Luminex 200 analyzer with Luminex 100 IS software. Assay sensitivity was 0.54 pg/mL for TNFα, 0.18 pg/mL for IL-1b, 0.28 pg/mL for IL-2, 2.54 pg/mL for IL-4, 0.31 pg/mL for IL-6, and 0.24 pg/mL for IL-10.

ELISA assay

Serum BAFF and TGFβ levels were tested using Human BAFF/BLyS/TNFSF13B Quantikine ELISA kit and Human TGFβ Quantikine ELISA kit (R&D Systems), following manufacturer protocol. Assay sensitivity was 0.191 pg/mL for TGFβ and 6.44 pg/mL for BAFF.

Alloantibodies

Anti-HLA antibody presence was confirmed with Luminex-based solid phase assays (SPA) using both screening and single-antigen tests. To reduce IgM interference, samples were frozen, then thawed before testing. Sera were screened for HLA-antibodies against class I and class II using a LABScreen Mixed bead assay (OneLambda Inc, Canoga Park, CA). Positively screened samples were further assessed with a LabScreen Single Antigen bead assay (One Lambda) to identify and classify anti-HLA antibodies as DSA (donor-specific antibodies) or non-DSA based on donor HLA typing (HLA-A, -B, -DR). Results exceeding 1000 MFI (normalized background formula) were recognized as positive. Anti-HLA antibodies were tested every 4 months during follow-up. The C1qScreen assay (One Lambda) was used to detect C1q-binding antibody.

Statistics

All between-group comparisons were made using the nonparametric Mann-Whitney U test, while Kruskall-Wallace tests were applied for multiple data sets. Relationships between two data sets were analyzed with Spearman rank correlations. Kaplan-Meier analysis was used to determine survival curves; between-group differences in survival were identified with a Cox-Mentel log-rank test and Gehan-Breslow-Wilcoxon test. Heat maps and cluster analysis was performed in ClustVis.[5] Data were presented as medians with interquartile range and visualized with bar graphs. The tops of each bar indicate means, while lines represent 95% confidence intervals. Significance was set at $p < 0.05$.

Table S1. Initial differences in measured parameters between Abs- and Abs+ recipients before transplantation

	**Abs-** n = 45, median value	**Abs+** n = 7, median value	**p (U test)**
CD19 [cells/µL]	111	107	0.77
CD5+ B cells [%]	9.27	12.93	0.20
Memory B cells [%]	20.83	17.61	0.41
Tregs [%]	3.11	5.06	0.00
BAFF [pg/mL]	839.47	868.48	0.34
IL-10 [pg/mL]	2.59	5.17	0.04
TGFβ [pg/mL]	16501.90	20924.62	0.91
IL-4 [pg/mL]	44.58	34.21	0.62
IL-1b [pg/mL]	1.69	1.60	0.43
IL-6 [pg/mL]	5.58	4.37	0.75
TNFα [pg/mL]	25.34	26.62	0.48

Gray highlighting indicates significant differences ($p < 0.05$).

Tregs - regulatory T cells

BAFF - B-cell activating factor

Figure S4. Memory B lymphocyte count at 2 y post-transplant

Percentage of total memory B cells in Abs- (open circles) and Abs+ (black circles) patients (n = 52) were compared for each measured time point, from transplant to 2 y post-transplant. *p < 0.05 based on U tests. The ratio of Abs- to Abs+ recipients at measured time points was as follows: transplant (45/7), +4 months (42/9), +8 months (40/12), +12 months (37/14), +16 months (35/15), +20 months (33/17), and +24 months (32/19).

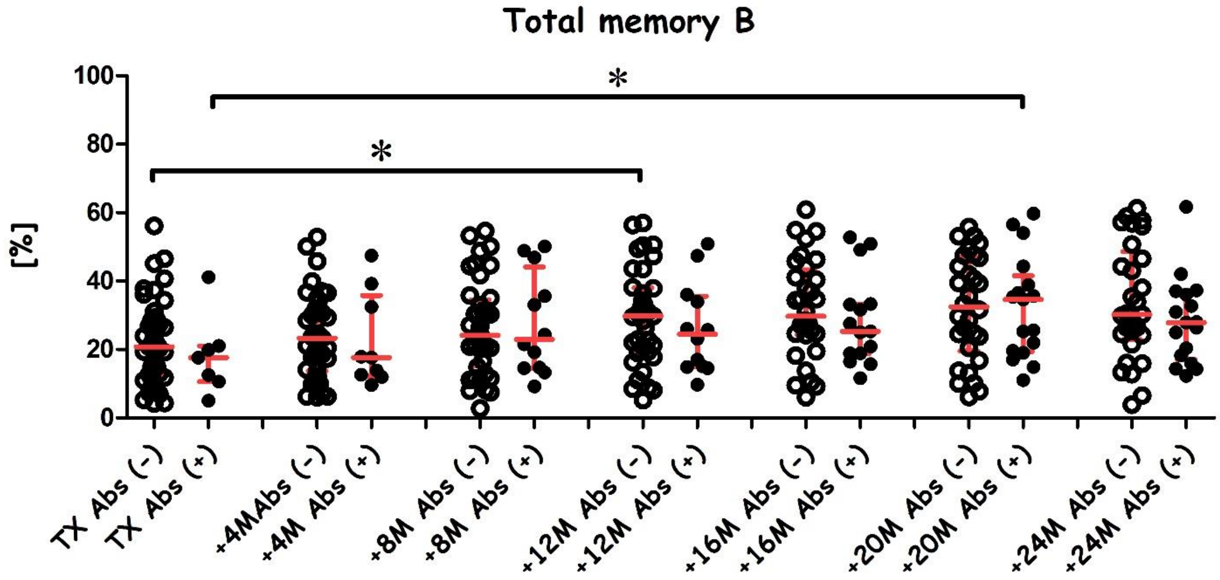

Figure S5. Number of recipients developing alloantibodies at 2 y post-transplant

(A) Proportion of patients developing alloantibodies (show in black) and spectrum of alloantibodies in the study population (n = 52), assessed every 4 months post-transplant. *p < 0.05 based on Chi-squared tests. **(B)** a table with a number of alloantibodies and MFI levels in each recipient; with red squares positive FCXM (B cells only) result indicated

A

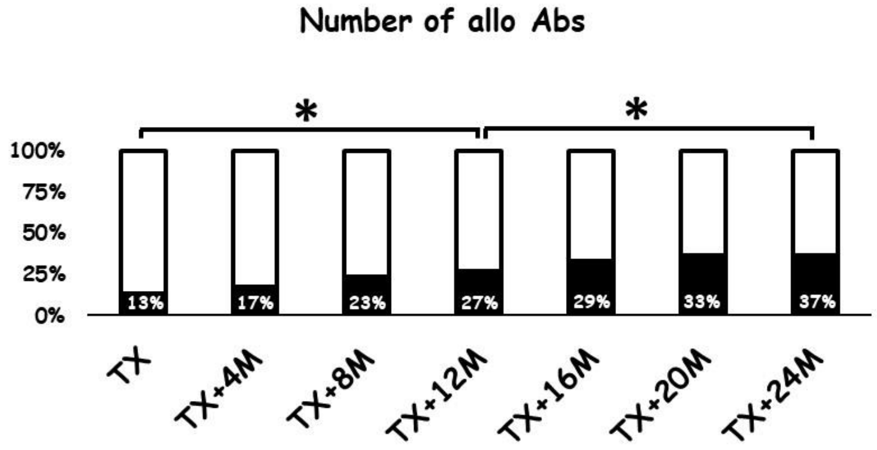

B

		TX				TX +4M				TX +8M				TX +12M			
No	Patient	Number of alloAbs	Number of DSA	Max MFI	Median MFI	Number of alloAbs	Number of DSA	Max MFI	Median MFI	Number of alloAbs	Number of DSA	Max MFI	Median MFI	Number of alloAbs	Number of DSA	Max MFI	Median MFI
1	5	1	0	1200		1	0	1000		1	0	2100		2	1	2200	1400
2	7	0				0				0				0			
3	13	1	0	2200		1	0	2600		1	0	2360		1	0	1890	
4	15	3	1	20000	10300	1	1	3200		1	1	3570		1	1	4020	
5	16	1	0	2000		1	0	2400		2	1	15000	10250	3	1	11000	3100
6	21	0				0				0				0			
7	23	0				0				0				0			
8	25	0				0				0				0			
9	27	1	1	5700		1	1	16800		1	1	12700		2	1	13610	6200
10	28	0				1	0	1000		1	0	1010		1	0	1000	
11	29	0				0				0				0			
12	30	0				0				1	0	1580		1	0	1400	
13	32	0				0				1	0	1110		2	0	1200	1000
14	33	0				0				0				1	0	1600	
15	35	1	0	3100		1	0	3700		1	0	1450		1	0	1100	
16	42	1	1	10400		1	1	1000		1	1	1100		1	1	3600	
17	45	0				0				0				1	0	2330	
18	47	0				0				1	0	1000		1	0	1100	
19	51	0				1	0	1900		1	0	1750		1	0	1360	
Number of (Abs+) patients		7				9				12				14			
Number of (DSA+) patients		3				3				4				5			
Number of (dnDSA+) patients		0				0				1				2			

		TX +16M				TX +20M				TX +24M			
No	Patient	Number of alloAbs	Number of DSA	Max MFI	Median MFI	Number of alloAbs	Number of DSA	Max MFI	Median MFI	Number of alloAbs	Number of DSA	Max MFI	Median MFI
1	5	1	1	1540		1	1	1300		2	1	2400	1100
2	7	0				0				1	0	1600	
3	13	1	0	2170		1	0	1700		1	0	1390	
4	15	2	1	4550	1170	2	1	3810	1650	2	1	3160	2240
5	16	3	1	8700	3300	1	1	1500		1	1	2300	
6	21	1	0	3600		1	0	2700		1	0	1900	
7	23	0				1	0	1000		1	0	1050	
8	25	0				0				1	0	1280	
9	27	1	1	15340		2	1	14500	7700	1	1	11830	
10	28	1	0	1200		1	0	1130		2	1	1200	1000
11	29	0				1	0	3990		1	1	3540	
12	30	1	0	1000		1	0	1000		1	0	1600	
13	32	2	0	1300	1000	2	0	1200	1010	2	0	1380	1020
14	33	1	0	1580		1	0	1590		1	0	1320	
15	35	1	0	1820		1	0	1960		1	0	1890	
16	42	1	1	3840		1	1	3500		2	1	3600	2010
17	45	1	0	1900		1	0	1720		1	0	1800	
18	47	1	0	1000		1	0	1060		1	0	1000	
19	51	1	0	1800		1	0	1800		1	0	1600	
Number of (Abs+) patients		15				17				19			
Number of (DSA+) patients		5				5				7			
Number of (dnDSA+) patients		2				2				4			

Figure S6. IL-4 levels at 2 y post-transplant

Serum IL-4 levels in Abs- (open circles) and Abs+ (black circles) patients (n = 52) were compared for each measured time point. *$p < 0.05$ based on U tests. The ratio of Abs- to Abs+ recipients at measured time points was as follows: transplant (45/7), +4 months (42/9), +8 months (40/12), +12 months (37/14), +16 months (35/15), +20 months (33/17), and +24 months (32/19).

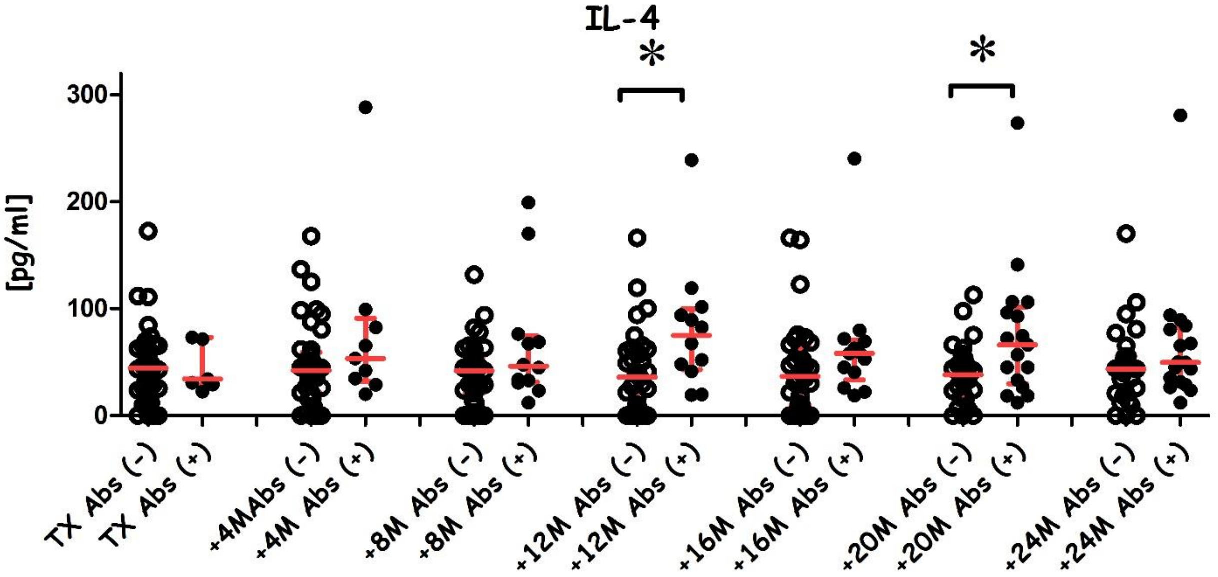

Figure S7. Rate of delayed graft function (DGF)

Proportion of patients developing DGF (in black) among the study population (n = 52). NS, not significant at $p < 0.05$ (Chi-squared tests).

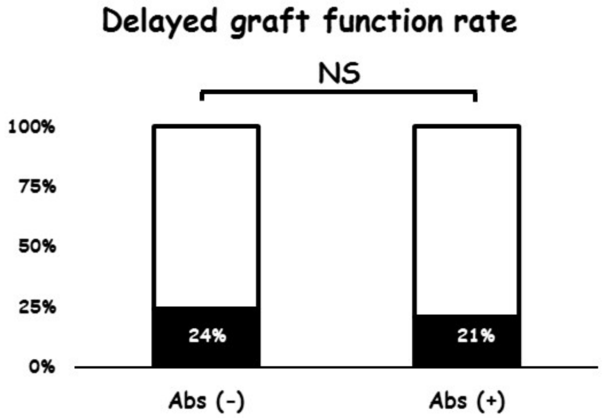

Figure S8. Patient survival rate at 8 y post-transplant

Patient survival rate was 75% for Abs- (n = 33) and 85% for Abs+ (n = 19) recipients. This difference was not significant based on both the log-rank test (p = 0.53) and Gehan-Breslow-Wilcoxon test (p = 0.45). The hazard ratio was 1.57 (95% CI: 0.46–5.32).

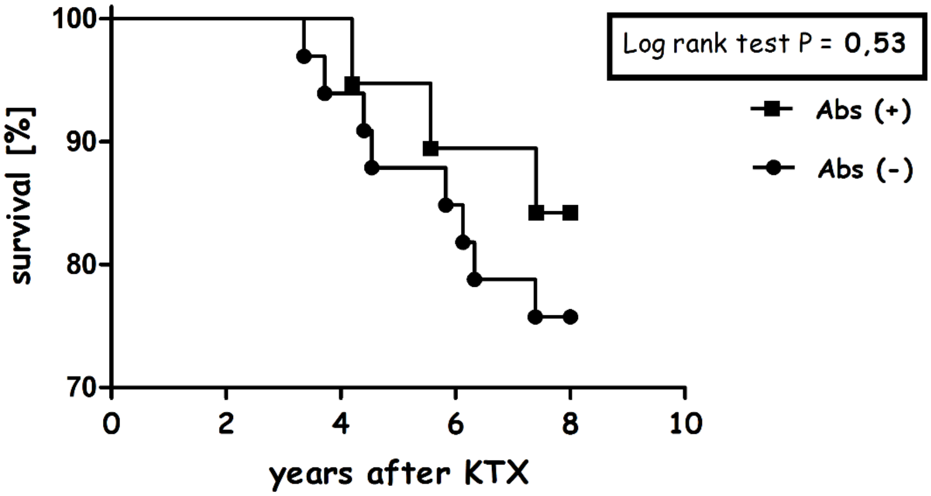

Figure S9. Neoplasm rate at 8 y post-transplant

Proportion of patients who developed skin neoplasm (in black) among the Abs- (n = 33) and Abs+ (n = 19) study populations. NS, no significant differences at $p < 0.05$ (Chi-squared tests).

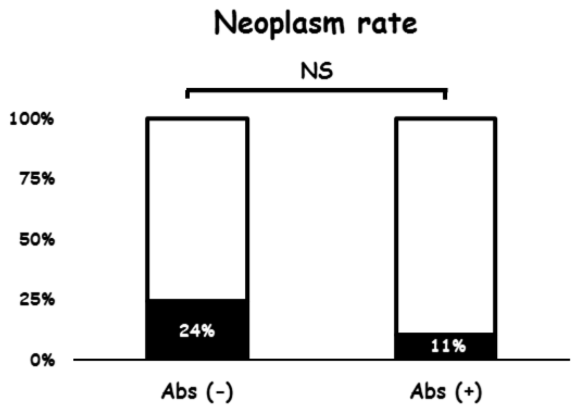

Figure S10. Serum concentrations of tacrolimus and cyclosporine after transplantation

Serum tacrolimus **(A)** and cyclosporine **(B)** levels in Abs- (open circles) and Abs+ (black circles) patients (n = 52) were compared 12 and 24 months post-transplant. NS, not significant at $p < 0.05$ (U tests).

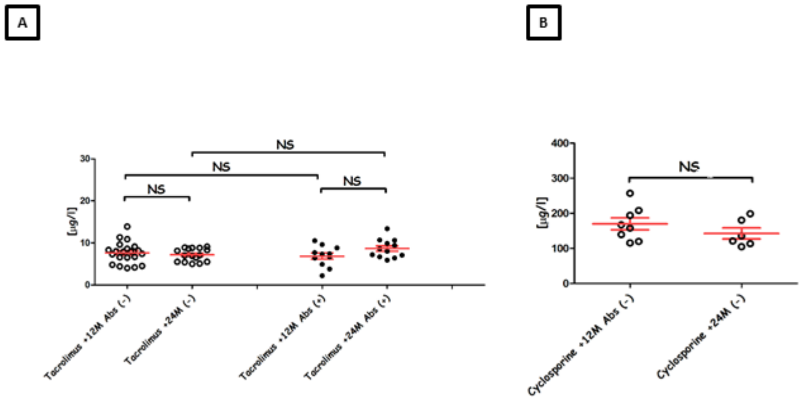

REFERENCES

1. Zasady Alokacji. http://www.poltransplant.org.pl/alokacja2.html. Accessed December 22, 2019.

2. Trzonkowski P, Szaryńska M, Myśliwska J, Myśliwski A. Ex vivo expansion of CD4(+)CD25(+) T regulatory cells for immunosuppressive therapy. *Cytom A*. 2009;75(3):175-188. doi:10.1002/cyto.a.20659

3. Trzonkowski P, Bieniaszewska M, Juścińska J, et al. First-in-man clinical results of the treatment of patients with graft versus host disease with human ex vivo expanded CD4+CD25+CD127- T regulatory cells. *Clin Immunol*. 2009;133(1):22-26. doi:S1521-6616(09)00697-4 [pii] 10.1016/j.clim.2009.06.001

4. Marek-Trzonkowska N, Myśliwiec M, Dobyszuk A, et al. Therapy of type 1 diabetes with CD4(+)CD25(high)CD127-regulatory T cells prolongs survival of pancreatic islets - results of one year follow-up. *Clin Immunol*. 2014;153(1):23-30. doi:10.1016/j.clim.2014.03.016

5. Metsalu T, Vilo J. ClustVis: a web tool for visualizing clustering of multivariate data using Principal Component Analysis and heatmap. *Nucleic Acids Res*. 2015;43(W1):W566-70. doi:10.1093/nar/gkv468
